# Aging in Time: Psychological and Behavioral Adaptations to Shifting Temporal Horizons

**DOI:** 10.1093/geroni/igaf122.343

**Published:** 2025-12-31

**Authors:** Yaeji Kim-Knauss, Frieder Lang, Yoav Bergman

## Abstract

Human aging unfolds within a dynamic temporal perspective that encompasses the past, present, and future. Lifespan development theories suggest that as individuals grow older, heightened awareness of life’s finitude influences psychological adaptation, motivation, and behavioral choices, which should in turn influence their well-being. Building on these theoretical foundations, five presentations will examine various dimensions of human experience shaped by shifting temporal perspectives. First, Nicole Long Ki Fung explores the trade-offs older adults make among pro-hedonic, pro-family, and prosocial goals as they adapt to shorter perceived time horizons. Expanding on the theme of goals and preferences, Claire Growney examines how appreciation of remaining time is linked to well-being and social preferences in older adulthood. Addressing the topic of coping and resilience, JoNell Strough investigates the mediating role of different time perspective in the relationship between age and emotional well-being, particularly in response to challenging life events such as natural disasters. Yaeji Kim-Knauss further examines how older adults experiencing health declines over time cope with mortality through two self-regulation strategies, namely, death preparation and death acceptance, and their respective effects on life satisfaction. Finally, highlighting the expression of shifting time perspectives, Talia Elkarif demonstrates how creative activities serve as a medium for older adults to articulate and construct evolving views of the future. In closing, Yoav Bergman will synthesize the key findings of the five talks, emphasizing different adaptive responses to limited temporal horizons in later life and their theoretical as well as practical implications.

